# Reliable synthesis of self-running Ga droplets on GaAs (001) in MBE using RHEED patterns

**DOI:** 10.1186/s11671-015-0890-7

**Published:** 2015-04-17

**Authors:** Beni Adi Trisna, Nitas Nakareseisoon, Win Eiwwongcharoen, Somsak Panyakeow, Songphol Kanjanachuchai

**Affiliations:** Semiconductor Device Research Laboratory, Department of Electrical Engineering, Faculty of Engineering, Chulalongkorn University, 254 Phyathai Road, Patumwan, Bangkok, 10330 Thailand

**Keywords:** Droplets, Sublimation, Self-running droplets, RHEED, MBE, GaAs

## Abstract

Self-running Ga droplets on GaAs (001) surfaces are repeatedly and reliably formed in a molecular beam epitaxial (MBE) chamber despite the lack of real-time imaging capability of a low-energy electron microscope (LEEM) which has so far dominated the syntheses and studies of the running droplets phenomenon. Key to repeatability is the observation and registration of an appropriate reference point upon which subsequent sublimation conditions are based. The reference point is established using reflection high-energy electron diffraction (RHEED), not the noncongruent temperature used in LEEM where temperature discrepancies up to 25°C against MBE is measured. Our approach removes instrumental barriers to the observation and control of this complex dynamical system and may extend the usefulness of many droplet-related processes.

## Background

Droplets on semiconductor surfaces play important roles in various devices and processes. Droplets have been used to enhance the conversion efficiency of solar cells through surface plasmons [1]; they also serve as efficient anti-reflection coating [2]. In the fabrication of nanoholes and nanowires, droplets are used as a drilling tool and a virtual template, respectively [3-5]. Through droplet epitaxy [6], droplets enable the fabrication of optoelectronic devices such as intersublevel infrared photodetector [7] and single-photon emitter [8]. The versatility of droplets can be increased further if droplet dynamics are better understood. Recently, a pioneering experiment involving Ga droplet dynamics on GaAs (001) was reported [9], stimulating further investigations in related systems [10-13]. These reports are conducted principally by *in situ* real-time observation under a low-energy electron microscope (LEEM), with limited availability, leading some to experiment using more readily available molecular beam epitaxial (MBE) chambers [14-16], albeit with limited yields since MBE is optimized for deposition, not for microscopy. It is now accepted that group III droplets nucleate and run on certain III-V surfaces undergoing sublimation, but many aspects of the self-running or self-propelled droplets remain unanswered [17]. With easier access and deposition capability, MBE has the potential to advance droplet dynamics studies with the ultimate aim of droplet controls in micro- and nanofabrication. Producing running droplets using MBE however is not trivial as inaccurate thermocouple temperatures often lead to under- or overdecomposition.

In this article, we report a simple procedure that leads to a reliable formation of self-running Ga droplets on GaAs (001) using *in situ* reflection high-energy electron diffraction (RHEED) patterns as the primary reference. Thermocouple temperatures serve only as rough indicators, secondary to the RHEED patterns. RHEED has been widely used for studying surface morphology during deposition [18-21]. But in this work, RHEED is used to predict the onset of the self-running droplets during decomposition. This method provides reproducible results of running Ga droplets in MBE which is important for those studying droplet imaging [22,23], dynamics [24], and control [25].

## Methods

All samples are scribed from epi-ready GaAs (001) wafers (AXT, Inc., Fremont, CA, USA). Each sample is attached to a molybloc and degassed at 450°C for 1 h. Afterward, the sample is loaded into Riber’s 32P MBE growth chamber and radiatively heated. The system pressure is kept below 5.5 × 10^−9^ Torr throughout. The samples then undergo a two-stage heating process: oxide desorption and sublimation. The first stage ramps the temperature of the substrate from room temperature at a rate not exceeding 30°C/min. The manipulator rotates at around a few rpm. During ramping, the set point temperature is put on hold whenever the chamber pressure approaches 5 × 10^−9^ Torr. After the pressure reduces below 10^−9^ Torr, set point ramping resumes. When the thermocouple temperature reaches 580°C, the ramp rate decreases to 10°C/min. Towards the end of the first stage, the oxide is removed and a streaky RHEED pattern appears. The sample manipulator is then rotated so that the electron beam from the RHEED gun impinges the sample in the $\left[1\overline{1}0\right]$ direction. The second stage ramps the temperature even more which results in a spotty RHEED pattern. The second stage is carried out without rotation. The streaky (spotty) pattern is associated with flat (rough) surfaces. For every significant change in the RHEED pattern, the heating is stopped, the sample is removed, and the surface morphology is studied by two microscopic techniques: optical microscopy (OM) with differential interference contrast (DIC) enhancement (Nikon’s Eclipse ME600P, Tokyo, Japan), and atomic force microscopy (AFM) using silicon nitride tips in the tapping mode in air (Seiko’s SPA400, Seiko Instruments, Tokyo, Japan). The two microscopic techniques allow meaningful correlation between RHEED patterns and surface morphology.

**Figure 1:**
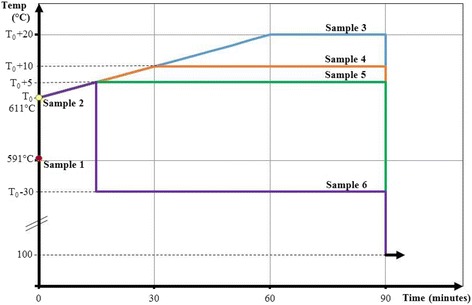
Temperature profiles of GaAs (001) samples sublimated in MBE.

The RHEED pattern from oxide desorption to sublimation of III-V surfaces evolve similarly: it slowly changes from streaky to spotty and matures to chevron, then fades away. Six samples are subject to different temperature profiles as shown in Figure 1. Controlled samples 1 and 2 show that the streaky and chevron patterns appear at thermocouple temperatures of 591°C and 611°C, respectively. Prolonged sublimation above the latter temperature results in the RHEED pattern disappearance, an expected result since the μm size droplets may scatter, absorb, or reflect the electron beam from the RHEED gun. RHEED pattern’s decay and disappearance is a characteristic typically associated with the growth of films with poor crystallinity or turning amorphous [26]. Thus, strictly, there is no direct information from the RHEED pattern to distinguish between a static, amorphous surface and one teeming with dynamic, running droplets. However, we are able to show that running droplets can be reliably formed simply by registering the chevron condition and applying appropriate temperature offsets and durations using appropriate profiles.

The chevron condition designates the temperature *T*_0_ where the chevron pattern appears. *T*_0_ serves as the reference temperature for the sublimation of samples 3 to 6 using the temperature profiles shown in Figure 1. Sample 3 is sublimated at *T*_0_ + 20°C for 30 min by ramping the temperature from *T*_0_ to *T*_0_ + 20°C at a rate of 0.3°C/min. This slow ramping rate allows droplet density control and prevents rapid decomposition [13]. Samples 4 and 5 are sublimated at *T*_0_ + 10°C and *T*_0_ + 5°C for 60 and 75 min, respectively, and ramped at the same rate as sample 3. Sample 6 is sublimated at *T*_0_ − 30°C for 75 min: the temperature *T* at first increases to *T*_0_ + 5°C to create a Ga-rich surface condition that stimulates Ga droplets nucleation, it then drops to *T*_0_ − 30°C and kept constant for 75 min. After quenching and sample removal, the surface morphology is studied by OM and AFM.

## Results and discussion

### Reference condition

**Figure 2:**
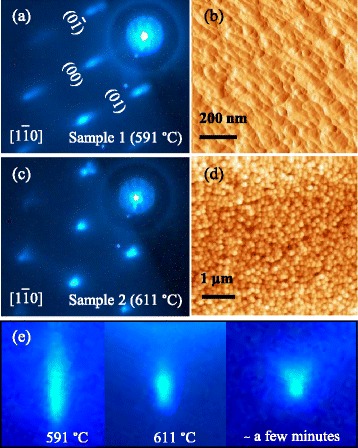
Correlation between RHEED patterns and surface morphologies. **(a)** The broad, streaky RHEED pattern during thermal desorption and **(b)** the corresponding AFM image showing surface corrugation after the initial deoxidation stage of sample 1 at 591°C. **(c)** The spotty/chevron RHEED pattern and **(d)** the corresponding AFM image showing nanoscale droplets formed during subsequent sublimation of sample 2 at 611°C. **(e)** Expanded RHEED images around the specular beam showing the evolution of the pattern from streaky at 591°C (left image) to spotty at 611°C (middle) which slowly transforms to the chevron pattern (right) when the substrate temperature is held constant. The chevron pattern develops at around *T*_0_ of 611°C, which serves as the reference temperature shown in the temperature profiles in Figure 1.

The reference condition is established with samples 1 and 2. Figure 2 shows the RHEED (left) and the corresponding AFM (right) images of the surfaces of samples 1 and 2. All sublimated samples undergo the condition of sample 1 with streaky pattern in Figure 2a and morphology in Figure 2b. These correspond to thermal desorption of native oxide which occurs at thermocouple temperature approximately 550°C to 590°C. As the temperature increases, all samples subsequently undergo the condition of sample 2, at *T*_0_, with spotty/chevron patterns in Figure 2c. These correspond to the early stages of noncongruent evaporation where excess Ga coalesce to reduce surface tension. As soon as the spotty pattern appears, the temperature is held constant (*T*_0_). Soon after, the spotty pattern sharpens and develops into a chevron pattern as shown by the evolution of the specular beam in Figure 2e; the latter is similar to those observed during epitaxial growth of quantum dots indicating the presence of facets [27]. The power supply to the substrate is turned off at this point and the sample is removed after the holder cools down to below 100°C. The surface is then probed by AFM, and the result in Figure 2d shows that the chevron pattern corresponds to nano-sized droplets with an average diameter and height of 20 and 30 nm, respectively.

These nanoscale droplets form spontaneously and homogeneously throughout the surface, giving extremely high droplet density. This characteristic is general in heteroepitaxy and does not require a nucleation layer [28]. A strong indication that the droplets seen in Figure 2d form spontaneously after oxide desorbed surface seen in Figure 2b is the root mean square (rms) roughness which increases from 5.7 nm in Figure 2b to 9.0 nm in Figure 2d. The nanoscale droplets later evolve into microscale droplets. For III-V (001) substrates, *in situ* LEEM experiments have confirmed homogeneous and spontaneous formation of micro- and nanoscale Ga and In droplets [12,13].

### Varying sublimation conditions

**Figure 3:**
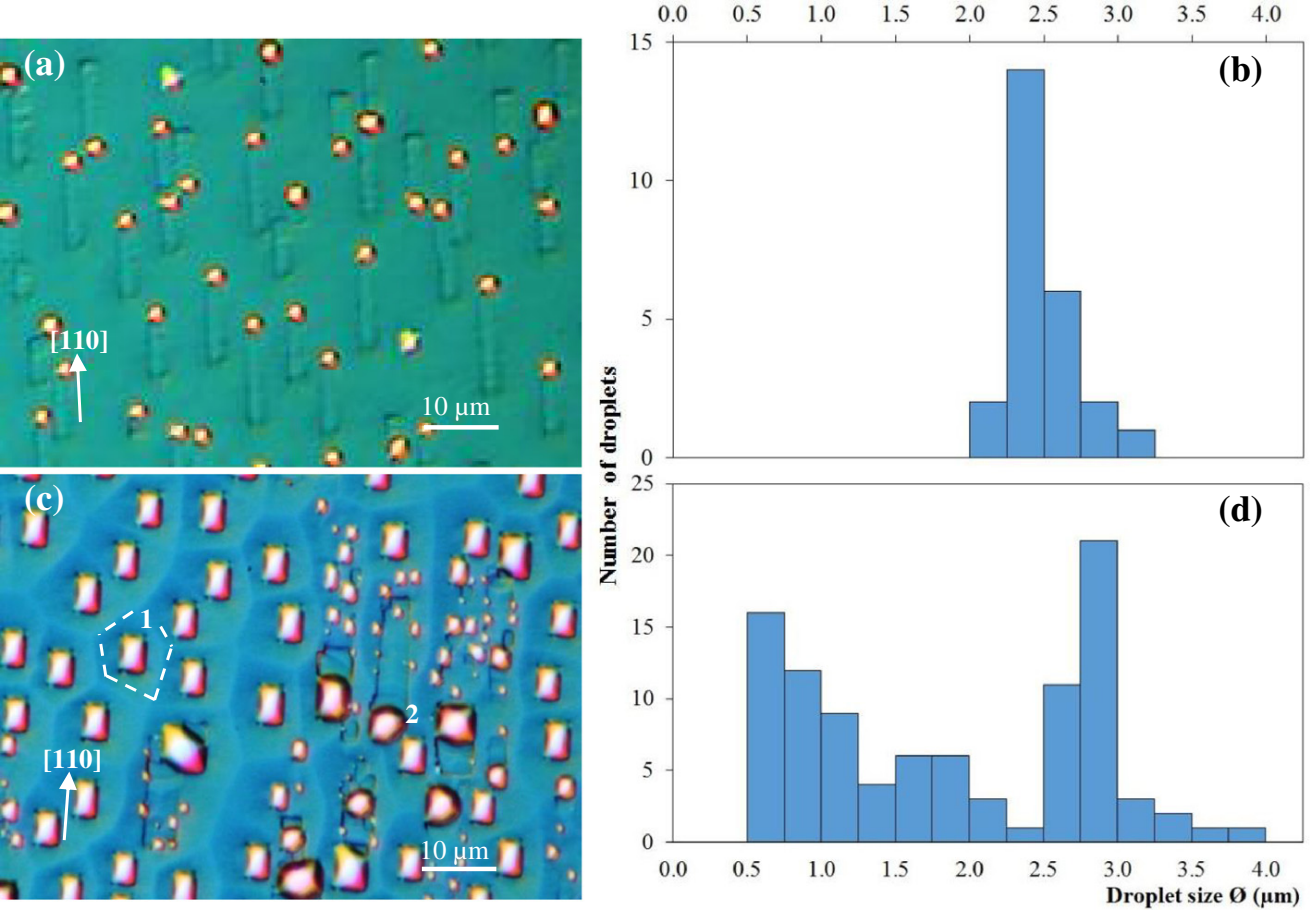
Surfaces of sample 3 after 30-min sublimation at *T*_0_ + 20°C. The sample suffers from temperature nonuniformity as **(a)** the DIC image and **(b)** the corresponding size histogram of the droplets in the cold zone differ significantly from **(c)** the DIC image and **(d)** the corresponding size histogram of the droplets in the hot zone.

Subsequent sublimation experiments exceed *T*_0_ and kept above *T*_0_ throughout for samples 3 to 5 or kept above *T*_0_ only momentarily for sample 6. Sample 3 is subject to the highest sublimation temperature (*T*_0_ + 20°C) and the sublimated surface is highly nonuniform. Figure 3a shows the DIC image of an area with small droplets with corresponding size histogram in Figure 3b. In contrast, Figure 3c shows the DIC image of another area with large droplets with corresponding histogram in Figure 3d. These illustrate surfaces at different stages of decomposition, most likely caused by temperature nonuniformity. The running droplet mechanism is highly sensitive to temperature and the rate of change of temperature: a slight variation in temperature, compounded by long sublimation time, could result in very different surface morphologies. The high degree of sensitivity is the main factor responsible for the small number of self-running droplet studies using MBE systems as they lack real-time, real-space imaging capability. Though temperature nonuniformity can generally be minimized using appropriate heater element and uniform backside radiation, the samples here are attached to the molybloc using indium (In) and thus a risk of nonuniformity is always present. Sample 3 is poorly prepared as unevenness of backside contact is clearly visible upon dismounting the sample from the bloc. The cloudy front areas - full of large, light-scattering droplets - are aligned with In-corrugated backside. Good thermal contact is achieved in these areas and hence they are referred to hereafter as the ‘hot’ zones. In contrast, the shiny front areas - populated by small droplets - are aligned with almost In-free backside. Poor thermal contact is achieved in these areas and hence they are referred to as the ‘cold’ zones. The droplets in the cold and hot zones differ both qualitatively and quantitatively.

The cold zones as exemplified by Figure 3a consist mainly of small, single-sized droplets. The Ga droplets have been running before quenching as the trails along the [110] direction consistent with earlier reports [9,12] are clearly visible. The histogram in Figure 3b shows that the droplet size is nominally 2.4 μm and falls within the 2 to 3.5 μm range. This supports the critical running size of 1.9 μm previously determined by Wu et al. [14]. These first-generation droplets are typically referred to as primary or mother droplets [16].

The hot zones as exemplified by Figure 3c are populated by large and small droplets. The droplet size distribution in this area as summarized in the histogram in Figure 3d shows that the droplets are bimodal: the large, primary droplets are nominally 2.9 μm and range from 2.4 to 4 μm in diameter, whereas the small, secondary droplets are as small as 0.5 μm. The surface is characteristic of late-stage coalescence [29] and represent the majority of reported sublimated III-V surface studies [30,31] before the realization of the self-running Ga droplets [9]. The running trails are either not formed or obliterated as a result of droplet coalescence. The absence of the running trails is associated with samples which have been sublimated at too high a temperature too quickly [13] where high-density droplets compete for material, delaying all to reach the critical running size. The droplets thus grow by coalescence. Once reaching the critical size, a droplet may be immobile or mobile depending on the surrounding. The droplet marked 1 in Figure 3c is immobile. The boundaries (shown as dashed lines) surrounding the droplet fix the droplet in place, blocking it from lateral motion. These boundaries do not exist in the cold zones as the droplets have plenty of space to move around. The droplet marked 2, on the other hand, is mobile due to the absence of nearby boundaries.

The shapes of these two groups of droplets are different: the immobile droplets are rectangular while the mobile droplets are curved, almost circular for some droplets. The rectangular shape is the original shape of the droplets due to the {111} bounding planes intersecting the (001) surface along the [110] and $\left[1\overline{1}0\right]$ directions, i.e., at right angles [12]. The rectangular droplets become more circular as they slip and hence less confined by the {111} planes. After slipping, the droplets gain more mass, etch the surface, and are again bound by the slow-etching {111} planes. The droplets’ stick-slip motion causes shape cycling as reported by Shorlin et al. [30]. The presence of the hot and cold zones on a 1″ area of a highly conductive solid sample shows that the running droplet phenomenon is highly temperature sensitive and explains why the phenomenon was not first detected in MBE. Due to this temperature sensitivity, subsequent samples were carefully mounted to ensure even distribution of In glue.

**Figure 4:**
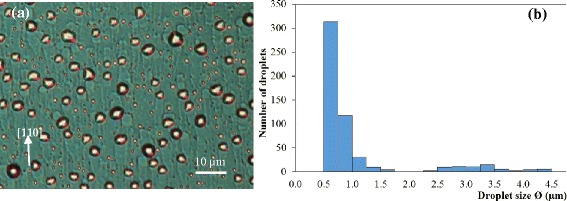
Sample 4 after 60-min sublimation at *T*_0_ + 10°C. **(a)** The DIC image and **(b)** the corresponding size histogram of the droplets on the post-sublimated surface.

**Figure 5:**
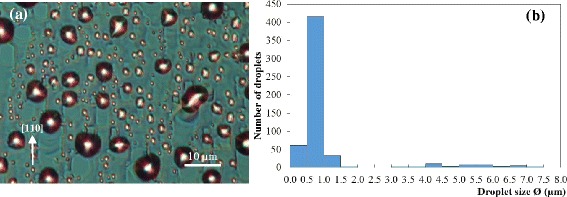
Sample 5 after 75-min sublimation at *T*_0_ + 5°C. **(a)** The DIC image and **(b)** the corresponding size histogram of the droplets on the post-sublimated surface.

Samples 4 and 5 are sublimated at lower temperatures but at longer durations than sample 3. Due to even distribution of backside In glue, post-sublimated surfaces of samples 4 and 5 are highly uniform. The DIC images and size histograms of sample 4 in Figure 4 and sample 5 in Figure 5 are thus representative of the whole samples and show that for both surfaces, small droplets exist in greater proportion than large droplets. The running trails are clearly evident in both cases. The DIC image of sample 4 shows that large droplets with diameter as wide as 4.5 μm exist on the surface whereas the largest droplets of sample 5 is approximately 7.5 μm. Since sample 5 is sublimated at a slightly lower temperature (5°C) but for longer (15 min) than sample 4 and that 5°C is within experimental accuracy (the uncertainty in visually registering *T*_0_ from sample to sample), the larger droplets in sample 5 are attributed to the longer sublimation period. The histograms further show that intermediate size droplets (2 to 4 μm) from sample 4 are significantly reduced in sample 5. These indicate that at 5°C to 10°C above *T*_0_, large Ga droplets grow at the expense of smaller droplets, a characteristic of Ostwald ripening.

The very large and very small droplets pose limits to applications in micro- and nanotechnologies, respectively, and it is thus important to know how they originate or evolve. Judging from the running trails, the largest droplets can occur as a result of the coalescence of two or more droplets running in opposite directions. Immediately after coalescence, the liquid droplets will try to distribute material so as to achieve the lowest surface energy but material mobility is limited by etched walls and thus the hemispherical shape of the droplets is unlikely for these large droplets. The small droplets, on the other hand, emerge from the walls of the etched trails; their presence as secondary droplets has been previously identified and studied in detail [16].

**Figure 6:**
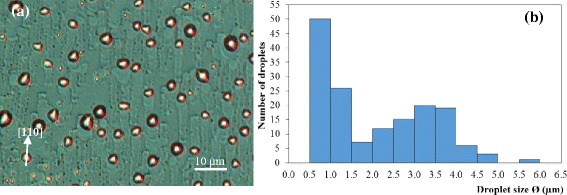
Sample 6 after 75-min sublimation at *T*_0_ − 30°C. **(a)** The DIC image and **(b)** the corresponding size histogram of the droplets on the post-sublimated surface.

Sample 6 is sublimated at the lowest temperature (*T*_0_ − 30°C) but for the longest time (75 min). The DIC image in Figure 6a shows that the average size of droplets is the smallest among all the sublimated samples, excluding those on the cold zones of sample 3. In addition, the histogram in Figure 6b indicates that sample 6 has very small number of small droplets (<1.5 μm). The absence of very small droplets possibly results from droplets shrinking while dwelling at a temperature lower than the congruent temperature *T*_C_ [9]. These results are consistent with expectations from thermal activated processes and are a strong indication that *T*_0_ − 30°C is less than *T*_C_. From the trail length, the average droplet velocity can also be estimated at 10 to 15 μm per hour.

### Surface and RHEED evolutions

Based on the size distributions and varying temperature profiles of the six samples, the relationship between the RHEED patterns and the evolution of the self-running Ga droplets on GaAs (001) can be established as follows. Starting from ramping up the temperature of the epi-ready GaAs (001), the first RHEED pattern observed is a broad, streaky pattern corresponding to corrugated surface as a result of thermal desorption of native oxides which occurs at *T*_deox_ of approximately 580°C. If the temperature ramping continues, the RHEED pattern changes from the broad, streaky appearance to one of spotty and chevron-like at *T*_0_, similar to those observed during growth - e.g., of InAs on GaAs. At this stage, the surface is roughened and becomes nonstoichiometric. This surface is associated with a Ga-rich, or equivalently an As-deficit, condition and denotes the first detectable sign of noncongruent evaporation in MBE. The temperature where the chevron pattern appears, or the chevron temperature *T*_0_, is not the same as the literature value of the noncongruent temperature *T*_C_ which in the case of GaAs (001) is 625°C [9]. *T*_C_ is the start of the noncongruent evaporation whereas *T*_0_ represents the condition at which nanoscale Ga droplets have already been formed. It is thus natural to assume that *T*_0_ is *higher* than *T*_C_. Our results, however, indicate that *T*_0_ is merely 20°C above *T*_deox_. Since *T*_deox_ is approximately 580°C, *T*_0_ is thus approximately 600°C, which is even *lower* than the literature value of *T*_C_ by as much as 25°C. For the self-running Ga droplets on GaAs (001), a temperature miscalculation of such magnitude - often the case without accurate temperature reference - would mean either overly sublimated or no sublimation conditions.

Sublimation above *T*_0_ results in the RHEED pattern quickly disappearing. The RHEED pattern as a guide to sublimation studies in MBE ceases to be useful at this point. Beyond this, systematic variations in sublimation time and temperature (with respect to *T*_0_) can instead be used to meaningfully produce and thus interpret the dynamics of group III droplets in MBE.

In MBE, it is very difficult to produce self-running droplets without RHEED. Prior to the six samples reported above, we failed to produce any self-running droplets in MBE despite having reliable temperatures from previously reported values of *T*_*C*_ which are mostly derived from LEEM experiments or taken from the literature value [9]. We achieved either very low- or very high-density droplets as a result of under- or oversublimation. No running trails are observed for both conditions. It is only after the proper reference temperature (*T*_0_) is established *in situ* (RHEED patterns) that self-running droplets are produced repeatedly. Many past experiments that sublimated III-V surfaces in vacuum failed to produce self-running III droplets mainly because the temperature or the pressure is too high; the former results in coalesced droplets [29] whereas the latter results in droplets etching instead of running [32].

## Conclusions

A simple procedure based on RHEED patterns is introduced and shown to be able to reliably produce self-running Ga droplets on GaAs (001) undergoing sublimation in an MBE chamber. Instead of relying on reported temperatures from other systems, the procedure registers the reference temperature *T*_0_ and systematically vary the sublimation time and temperature around *T*_0_ to achieve the running droplets on all samples tested. While III-rich surface conditions have been known to the MBE community for decades, the self-running III-droplets have only recently been discovered using *in situ* microscopy [9]. The temperature sensitivity of the running droplet mechanism results in very few instances of this type of studies conducted in MBE [16]. Now with our proposed procedure, the running droplet mechanism can be easily accessed and probed, paving the way for improved fundamental understanding of this rarely reported mechanism and of dynamics at liquid-solid interfaces in general.

## Abbreviations

AFM: atomic force microscopy
DIC: differential interference contrast
LEEM: low-energy electron microscope
MBE: molecular beam epitaxy
OM: optical microscopy
RHEED: reflection high-energy electron diffraction
